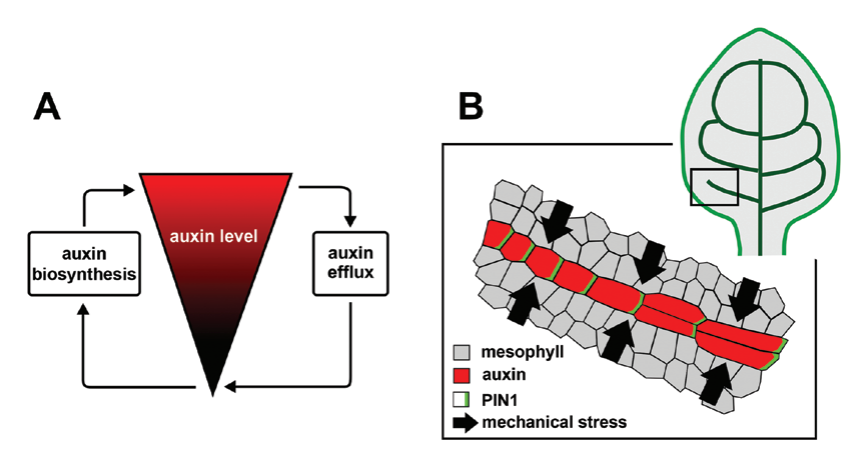# Shaping leaf vein pattern by auxin and mechanical feedback

**DOI:** 10.1093/jxb/eraa499

**Published:** 2021-02-24

**Authors:** Agata Burian, Magdalena Raczyńska-Szajgin, Wojtek Pałubicki

**Affiliations:** 1 Institute of Biology, Biotechnology and Environmental Protection, University of Silesia in Katowice, Katowice, Poland; 2 Faculty of Mathematics and Computer Science, Adam Mickiewicz University, Poznań, Poland

**Keywords:** Auxin, leaf, mechanical stress, vascular system

## Abstract

This article comments on:

**Kneuper I, Teale W, Dawson JE, Tsugeki R, Katifori E, Palme K, Ditengou FA**. 2021. Auxin biosynthesis and cellular efflux act together to regulate leaf vein patterning. Journal of Experimental Botany **72**, 1151–1165.

**Auxin is an essential factor for the specification of veins in plant organs. The distribution of auxin in tissues depends on several physiological processes including auxin biosynthesis and transport. By using empirical data and theoretical analysis,** Kneuper *et al.* (2021) **explored a role for these processes in the establishment of leaf vasculature in Arabidopsis. They propose that the formation of early vein patterns may essentially be described in terms of auxin biosynthesis, its transport, and growth-dependent mechanical feedback from surrounding tissues.**

Multicellular organisms need an integrative system enabling fast and effective transport of substances and communication between distant parts of their body. This function is fulfilled by the vascular system, the formation of which is strictly related to the developmental programme of each organ. In plants, the phytohormone auxin plays the role of an instructive signal in the establishment of the vascular system. In his pioneering studies, Tsvi Sachs (1981) proposed that the formation of vascular strands is initiated by the directional movement of auxin from a source to a sink which is gradually restricted (or canalized) into narrow files of cells. Later, it was proposed that the directional auxin movement is mainly determined by PIN proteins which are polarly localized in the plasma membrane and act as auxin efflux carriers (Palme and Gälweiler, 1999).

Although the reduction of polar auxin transport either by chemical treatment or by mutations does not prevent the formation of veins, their pattern is altered compared with non-treated or wild-type (WT) plants, respectively (Verna *et al.*, 2019). For example, a striking feature of 1-*N*-naphthylphthalamic acid (NPA)-treated plants is a fan-like pattern of leaf vasculature where a primary vein (midvein) consists of numerous parallel strands which extend towards the leaf margin and form a continuous vascular domain along the margin (Box 1B). Because the reduction of polar auxin transport may hamper auxin drainage from the source, NPA-induced extensive vascularization, manifested in higher vein density, may be explained in terms of the accumulation of auxin at sites of its production (Rolland-Lagan and Prusinkiewicz, 2005).

Box 1. **Compensation of reduced auxin biosynthesis by reduction of auxin efflux**

Wild-type leaf vasculature forms a continuous branched system where higher order veins diverge from the primary vein and frequently connect with other veins, making loops (A). The reduction of auxin efflux by NPA treatment leads to an increased number of veins, increased vein thickness, and a fan-like pattern (B). Conversely, a decreased number of veins that form discontinuous pattern, missing the above-mentioned loops, develops when auxin biosynthesis is reduced by genetic disruption of the TAA/YUCCA pathway, or by treatment with kynurenine (an inhibitor of TAA activity) (C). Kneuper *et al.* (2021) found that these abnormal vein patterns can be rescued by simultaneous reduction of both auxin biosynthesis and auxin efflux (D). Accordingly, they proposed that vein formation depends on the relative strengths of these two processes.

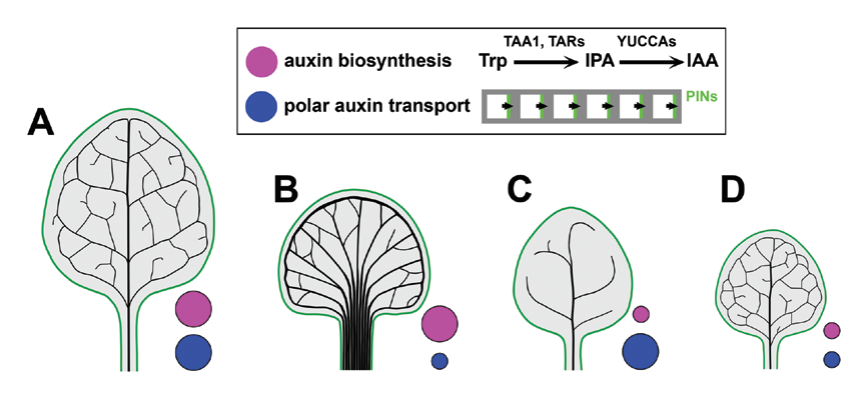

Auxin is predominantly synthesized from tryptophan in a two-step pathway involving TAA1/TAR aminotransferases and YUCCA flavin monooxygenases. Any disruption of this pathway, which significantly reduces free auxin levels, leads to a decrease in the number of veins and the generation of discontinuous vein patterns (Box 1C) (Cheng *et al.*, 2007; Stepanova *et al.*, 2008). Therefore, auxin transport and auxin biosynthesis are central regulators of vein patterning through their effect on auxin distribution in a tissue.

Testing interactions between auxin transport and biosynthesis, Kneuper *et al.* (2021) found that an abnormal vein pattern which is formed due to genetic or chemical reduction of auxin biosynthesis can be rescued by NPA-induced reduction of polar auxin transport (Box 1). This implies that reduced auxin production can be compensated by reducing auxin efflux. Importantly, this compensation seems to be specific for vein patterning, but not for other auxin-dependent processes, such as leaf growth (Kneuper *et al.*, 2021) or organogenesis (Cheng *et al.*, 2007).

## Computer simulation of vein formation

A substantial amount of experimental, as well as theoretical evidence suggests that the formation of veins depends on so-called canalization of polar auxin transport (reviewed in Ravichandran *et al.*, 2020). In contrast, Kneuper *et al.* (2021) propose a mechanism that does not depend on canalization at all. Instead, they show that early stages of vein patterning (i.e. a midvein formation) can be achieved via a simple biomechanical signaling feedback between auxin-synthesizing cells (future vascular cells) and the surrounding tissue. Specifically, Kneuper *et al.* (2021) use computer simulations of auxin dynamics in 2D representations of young leaf geometry. Their model is based on the following assumptions: (i) cell growth is inversely proportional to the net forces acting on cell walls; and (ii) growth of cells that do not synthesize auxin is proportional to auxin concentration. Further, the authors propose an analytical solution to a set of differential equations that express the above assumptions. This solution is a mathematical function of space and time that describes relationships between auxin synthesis rate, cell growth rate in area, and auxin diffusion rate. Essential to this model is the effect of auxin-synthesizing cells to promote growth of surrounding cells and inhibit their own proliferation via resulting compressive stresses. Kneuper *et al.* (2021) show that such mechanical feedback may enable the emergence of narrow cell files in a midvein. Their model also describes the abnormal midvein shape after NPA treatment alone and the rescue from this effect in auxin-deficient plants.

The concept of biomechanics as a signal for cell growth and vein patterning has been discussed in a number of studies. Previous models used biomechanical signaling to explain vein patterns in terms of tissue-scale compressive stress of the mesophyll, the orientation of cell division planes, or altered mechanical properties of cell walls (Couder *et al.*, 2002; Laguna *et al.*, 2008; Corson *et al.*, 2009; Lee *et al.*, 2014). Here, Kneuper *et al.* (2021) postulate that compressive stress distinguishes regions of auxin production (future vascular cells) from the rest of the leaf tissue, leading to their specific growth dynamics. However, unlike previous approaches, the model is confined only to the very early stages of vasculature formation.

## Localized auxin biosynthesis in leaf primordia

There exists a controversy on auxin concentration in pre-existing veins. On the one hand, experimental data invariably show the activity of many auxin-related genes, implying higher auxin concentration in pre-procambial and procambial cells compared with surrounding cells (Scarpella *et al.*, 2006). On the other hand, theoretical models predict that high auxin flux can lead to low auxin concentration in pre-existing veins (Rolland-Lagan and Prusinkiewicz, 2005). However, high auxin concentration in pre-existing veins is made plausible by the implementation of additional factors, such as dynamics of auxin flux and efflux carriers (Feugier *et al.*, 2005), or a modulation of the source strength (Feller *et al.*, 2015). High auxin concentration in pre-existing veins may also be maintained by *in situ* auxin biosynthesis. In fact, Kneuper *et al.* (2021) show that expression domains of TAAs and YUCCAs overlap not only at the primordium margin, but also at sites of vein formation, suggesting that auxin production is localized in pre-existing veins. Thus, localized auxin biosynthesis would explain high auxin concentrations in cells with high auxin flux.

Such localization of auxin biosynthesis, however, imposes further questions. How is auxin biosynthesis spatially regulated? Is there a link between auxin biosynthesis and PIN1-mediated auxin efflux? In agreement with previous studies (Suzuki *et al.*, 2015), Kneuper *et al.* (2021) show that YUCCA genes are down-regulated after the application of exogenous auxin. Conversely, YUCCA genes are up-regulated when auxin levels are reduced by kynurenine (Suzuki *et al.*, 2015). These data indicate a negative feedback between auxin levels and auxin biosynthesis. Since PIN1 may reduce auxin levels in the protoplast by moving auxin into the apoplast, it could be expected that auxin biosynthesis is regulated by PIN1-mediated auxin efflux. As a matter of fact, the expression of YUCCA genes has been found in leaf boundaries where low auxin levels are predicted due to a divergent pattern of PIN1 polarity (Abley *et al.*, 2016). Therefore, these data provide good arguments that localized auxin biosynthesis could balance auxin drainage due to polar auxin transport in pre-existing veins (Box 2A). Furthermore, as PIN1 expression can be induced by auxin (Heisler *et al.*, 2005), auxin biosynthesis in pre-existing veins could also maintain the pool of PIN1 needed for auxin transport. However, it remains to be determined whether PIN1-dependent auxin efflux is sufficient to locally up-regulate auxin biosynthesis. Alternatively, other regulators could be involved, because TAA and YUCCA genes can be expressed independently of PIN1 activity (Kneuper *et al.*, 2021).

Summarizing, current empirical and theoretical studies show that the formation of vein patterns can be described in terms of auxin-dependent specification of future vascular cells and their ability to respond to biomechanical signals generated at the tissue level (Box 2). The study of Kneuper *et al.* (2021) highlights the importance of auxin level balance depending on auxin transport and its biosynthesis, which can become an exciting area for future studies on the vascular system in plants. Moreover, as the vein formation is intrinsically related to cell morphogenesis, it is still an open question how exactly auxin specification is translated into changes of cell shapes.

Box 2. **Vein formation by auxin and biomechanical signals**

Auxin-dependent specification of future vascular cells can depend on the interaction between polar auxin transport and auxin biosynthesis (A). After the specification, these isodiametric cells are transformed into strongly elongated procambial cells due to anisotropic growth and the specific orientation of the cell division plane (Nelson and Dengler, 1997) (B). Growth anisotropy of future vascular cells can be determined by mechanical stresses at the tissue level resulting from differential growth in surrounding tissues (Couder *et al.*, 2002; Laguna *et al.*, 2008; Corson *et al.*, 2009; Kneuper *et al.*, 2021). Mechanical stresses may also determine the specific orientation of the division plane in future vascular cells (Louveaux *et al.*, 2016). Furthermore, since the differentiation of vascular cells is related to chemical and physical modifications of their cell walls (e.g. lignification), differentiated veins may impose additional forces and modify the pattern of mechanical stresses.